# The influence of different morphological units on the turbulent flow characteristics in step-pool mountain streams

**DOI:** 10.1038/s41598-021-99564-7

**Published:** 2021-10-11

**Authors:** Sruthi Thazhathe Kalathil, Venu Chandra

**Affiliations:** Department of Civil Engineering, Indian Institute of Technology Madras, Chennai, 600036 India

**Keywords:** Hydrology, Restoration ecology, Geomorphology

## Abstract

The morphology of step-pools is often implemented for ecological restoration and for the creation of close-to-nature fish passes. Step-pools display spatio-temporal variations in bed and flow characteristics due to meso-scale units such as step, tread, base of step, and pool. Exclusive research on the effects of bed variations in step-pools on the flow dynamics is limited. Here, we conducted laboratory experiments on a physical model downscaled from a field site in the Western Ghats, Kerala, India. The results of Kruskal–Wallis ANOVA show significant differences in the velocity and turbulent intensities for the morphological units. A regression equation of the form Power-Allometric1 has been proposed to relate the normalized turbulent kinetic energy with the velocity magnitude. The present study also estimated the range of Reynolds shear stress and energy dissipation factor existent in the step-pool systems. The normalized values of Reynolds shear stress in the *x*–z plane ranged from − 19.477 to 13.729, and energy dissipation factors obtained for the three step-pool systems are 321, 207, and 123 W/m^3^; both the results reveal insufficient pool volume for adequate energy dissipation. The study concludes that while designing close-to-nature step-pool fish passes, pool dimensions should be finalized with respect to the target aquatic species.

## Introduction

**Figure 1:**
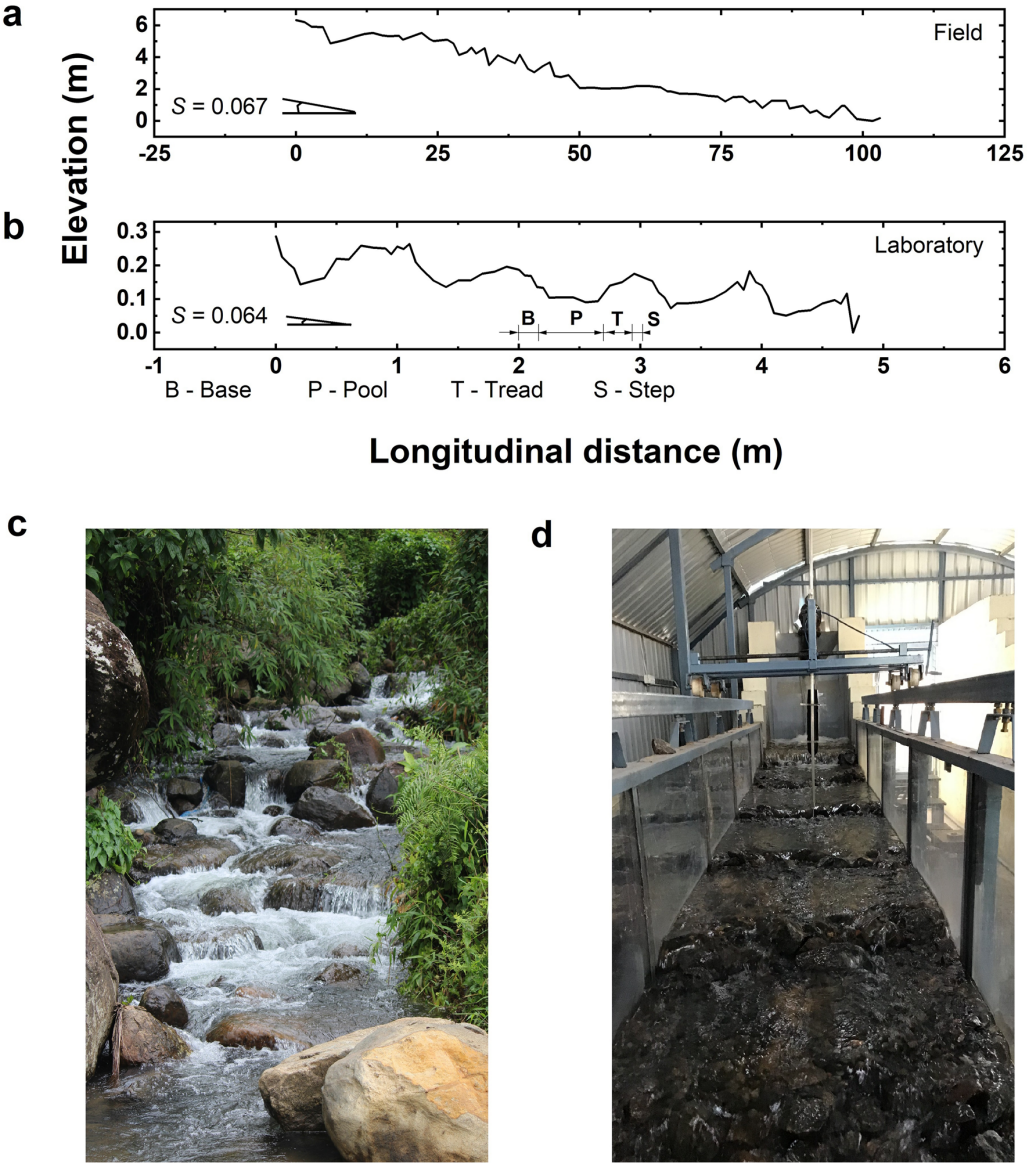
Details of step-pool systems in the present study: (**a**) Longitudinal section of the field site, (**b**) Longitudinal section of the laboratory model, (**c**) Photograph of the field site, (**d**) Photograph of the experimental setup.

Step-pools are natural geomorphologic forms developed under the action of extreme floods^1^ in mountain streams with bed slopes ranging from 3 to 20%^2,3^. The step-pools are characterized by poorly graded bed materials intricately packed to form a step and pool sequence, generating high energy tumbling and tranquil mountain flows. The typical bed morphology is irregular and results in spatially and temporally varied hydrodynamics over various functional units within the step-pools such as step, tread, base of step, and pool region (Fig. 1b). The step denotes the portion of bed comprising boulders or bedrock outcrops jam-packed across the width of the channel. The pool consists of finer bed materials and deeper cross-sections as a result of scour due to submerged or unsubmerged hydraulic jumps generated in the pool. The base of step is the section immediately downstream of step unit where the flow over the step impinges into the pool with high amounts of turbulence and self-aeration. The tread is the region extending from the downstream end of the scour pool up to the step unit. Step-pool systems can also exist without the presence of a tread region. In that case, the pool region directly ends in a step unit^4^.

The evaluation of flow parameters in step-pool streams does not follow the general criteria recommended for lowland rivers. The commonly used flow friction factors such as Manning’s *n* and Chezy’s *C* cannot be applied here due to the non-uniform nature of flow at meso-scale. In step-pool mountain streams, the rational frictional coefficient to define flow resistance is the non-dimensional Darcy Weisbach friction factor^5–7^. Dedicated field and laboratory investigations of the step-pools are necessary to create a sufficient database for the development of accurate hydraulic models.

In addition, the design of step-pools is adopted for stream restorations^8,9^, storm water conveyance systems^10^, and for creating close-to-nature step-pool fish passes^11–13^. Primal research on step-pools has been largely limited to the analysis of bed morphology^14–17^, flow resistance^18–22^ and sediment transport^23,24^ by considering the step-pool reach as a single system. A detailed review on the hydrodynamics of step-pools in mountain streams is available in Kalathil and Chandra^7^.

The variations in the flow characteristics imposed by the various morphological units within the step-pool system (SPS) were not studied until the 2000s. Adverse pressure gradients in the pools and upstream of steps lead to increased turbulence, while favourable pressure gradients on steps suppress turbulence. Accordingly, pools are dominated by wake turbulence and the steps, treads and runs are governed by form or bed-generated turbulence. The wake turbulence in pools is characterized by recirculation eddies and its strength diminishes with increase in distance from the impingement point^25,26^. Incidentally, the variations in hydrodynamics within step-pool systems do not furnish considerable differences in the sediment transport estimation since the measured and computed magnitudes differ up to an order of three because of the limited sediment availability in mountain streams^27,28^. Nevertheless, updated knowledge on the flow dynamics at different regions within the step-pool reach will aid in providing guidelines for designing close-to-nature fish passes to enable target species to pass through the fluvial system^29,30^. In recent times, with increased demands to implement and maintain environmental flow schemes, cost-effective and eco-friendly structures such as step-pools provide a promising tool to facilitate economic development together with ecological conservation.

The presence of a wide range of substrates (fine sand to boulders) and varying flow conditions in step-pools facilitate the inhabitation, migration, and dispersal of diverse aquatic species^31^. The productive range of water depth and flow velocity for inhabitation lies between 0.16 m to 0.5 m and 0.3 m/s to 1.2 m/s, respectively^11^. In addition to the range of flow depth and velocity requirements, hydraulic shear stress and turbulence characteristics also affect fish behaviour and locomotion^32^. Depending on the turbulence scale and intensities, various damages on the fish body or disorientation of the species may occur. Therefore, it is important to consider fish behaviour, life stage, swimming ability, and hydraulic conditions including velocity and turbulence characteristics in step-pool structures prior to its ecological applications. Adequate design guidelines for the construction of close to nature fish passes are not available due to lack of studies^31,33^.

Limited research addresses the influence of bed morphology on the turbulence characteristics in step-pools. Wohl and Thompson^4^ studied the variations in flow profiles at different locations in a step-pool with the use of an electromagnetic current meter of sampling frequency 0.5 Hz. The study was limited to the analysis of mean velocity and coefficient of variation in velocity which is sometimes used synonymous to turbulence intensities. Although flow profiles showed variations in pattern, ANOVA and ANCOVA results were rather inconclusive regarding the dependence of flow parameters on the bed form types. Later on, Wilcox and Wohl^34^ and Wilcox et al.^35^ conducted three-dimensional velocity measurements using SonTek FlowTracker operating at 1 Hz sampling frequency to study the spatial variation of velocity and turbulence intensities in step-pools. The pools exhibit increased levels of turbulence intensities and less velocity reduction in cases where the upstream step-units do not span the entire width of the channel and effects as leaky or porous steps^35^. The turbulence characteristics in terms of the root mean square of the fluctuating velocities showed considerable differences between step, tread and pool. However, due to the low sampling frequency of the velocity measuring instrument, the accuracy of turbulence analysis is questionable. Considering the complex terrain in step-pool streams and practical difficulties in the use of high frequency instruments that require proper stationing and continuous power supply, it is arduous to produce good quality data of the fluctuating velocities. To bridge the gap in research on the fluctuating velocity components in step-pools, extensive laboratory studies are required.

In this context, to shed light upon the variation of hydraulic parameters with the morphological units, we discuss the results from a physical model downscaled according to field measurements conducted in a step-pool stream in Erumakolli, Wayanad, India. Figure 1 shows the longitudinal sections and photographs of the field site and the laboratory experimental setup. The field investigation comprised the measurements of bed material size, bed topography and flow velocity measurements. The physical model study discusses the variation in the turbulence characteristics across steps, treads and pools. The analysis is limited to the vertical distribution of velocity magnitude and turbulence intensities across the morphological units, the propagation of velocity magnitude and Reynolds shear stress in the flow direction, relationship between the turbulent kinetic energy and velocity magnitude, and the evaluation of energy dissipation factor in the step-pools. The present study is the foremost attempt in the analysis and discussions of turbulence fluctuations, Reynolds shear stresses and energy dissipations in self-formed step-pool systems.

### Experimental setup validation

The laboratory experimental setup was created by establishing dynamic similarity between the field and the laboratory model through Froude’s Model Law. Model scales less than 10:1 can successfully simulate field conditions in the case of turbulent self-aerated flows^36^. A length scale ratio of 3.3: 1 was chosen for creating the physical model. The corresponding velocity scale and discharge scale are (3.3)^1/2^: 1 and (3.3)^5/2^: 1, respectively. The laboratory step-pool system is self-formed under a formative discharge and is not expected to generate the exact bed topography as observed in the field. However, effect of the influencing parameters such as *D*_84_, step-height, bed slope, and discharge on the velocity and turbulence characteristics would be adequately simulated. A comparison of the thalweg velocity and Froude number over step, tread and pool at *d* = 0.6 *H* between the field and laboratory data is presented in Table 1, where *d* is the depth of measurement and *H* is the total flow depth at that point. Since the measured data is location specific and due to the limitations in the number of data points available, only the reach scale average values of velocity and Froude number was used to estimate the error. An absolute error of 0.04 m/s (6.3%) and 0.02 (4.9%) was observed between the field and laboratory up scaled data for thalweg reach average velocity and Froude number, respectively.

**Table 1:** Comparison of the thalweg velocity and Froude number for step, tread and pool at *d* = 0.6 *H* between the field and laboratory data.

	N	Step			Tread			Pool			Reach Average		
		Min	Max	Avg	Min	Max	Avg	Min	Max	Avg	Min	Max	Avg
**Velocity Magnitude (m/s)**													
Field	9	0.57	1.4	0.92	0.71	0.97	0.81	0.12	0.33	0.2	0.12	1.4	0.64
Laboratory model	30	0.57	0.79	0.71	0.11	0.49	0.33	0.1	0.49	0.32	0.1	0.79	0.38
Laboratory data in prototype scale		1.03	1.44	1.28	0.2	0.88	0.61	0.18	0.89	0.58	0.18	1.44	0.68
**Froude number**													
Field	9	0.32	1.15	0.67	0.58	0.72	0.65	0.05	0.14	0.09	0.05	1.15	0.41
Laboratory model	30	0.72	1.27	1.04	0.11	0.65	0.38	0.09	0.49	0.32	0.09	1.27	0.43

*N* denotes the number of data points.

### Velocity and turbulence intensities

The velocity and turbulence characteristics pertinent to accurate design and model development of step-pools are velocity magnitude (*V*_*R*_), turbulence intensities (*TI*), normalized turbulent kinetic energy (*K*), and energy dissipation factor (*EDF*). We obtained velocity data in the physical model using Nortek Vectrino 3-D Acoustic Doppler Velocimeter. A total of 16 thalweg velocity data at *d* = 0.6*H* and 24 vertical velocity profiles at 1 cm intervals have been retained after velocity filtering and processing (see “Methods”), where *d* is the depth of measurement and *H* is the total flow depth at that point. The velocity measurements were confined in the range of 0.003 m/s to 0.796 m/s, bounding the productive range for aquatic species inhabitation in field scale (0.005 m/s to 1.446 m/s).

**Figure 2:**
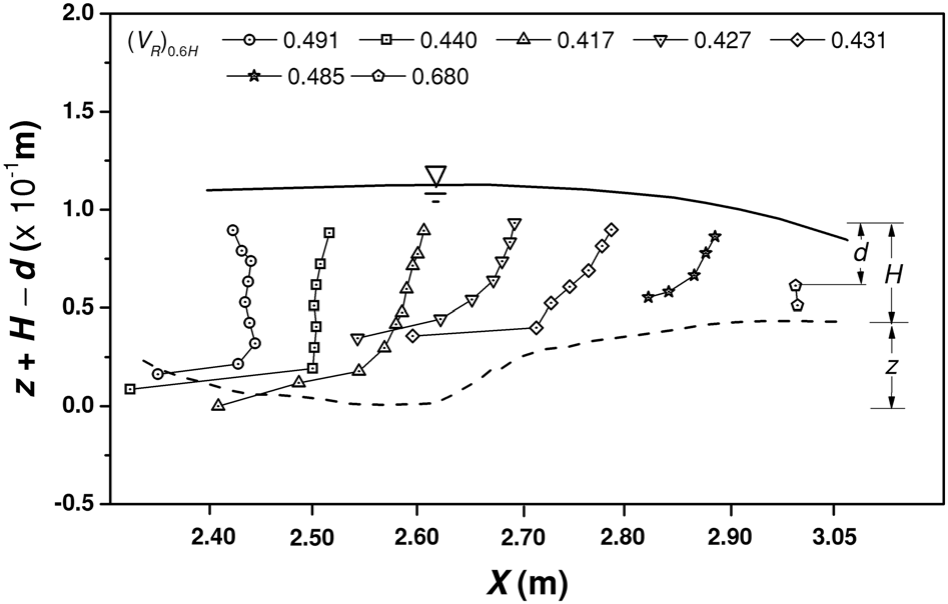
Variation of resultant velocity magnitude *V*_*R*_ in the longitudinal direction of the SPS 2.

The propagation of flow in a step-pool system is illustrated in Fig. 2. The *x*-axis shows the measurement sections along the longitudinal direction (*X*) for step-pool system 2 (see “Methods”). The variable on the *y*-axis *z* + *H* − *d* denotes the elevation of the measurement point above the datum which is set at the deepest scour point of *X* = 2.60 m. Where, *z* is the vertical distance from the datum to the bed surface, *H* is the total flow depth at the point, and *d* is the depth of measurement with respect to the free surface. The first vertical corresponding to *X* = 2.40 m is at a distance of 0.15 m downstream of a step unit. Any data collected closer to the steps were removed in data filtration. The average velocity at *d* = 0.6*H* is shown in the plot legend. The lowest velocity is observed at the deepest scour section of *X* = 2.60 m.

**Figure 3:**
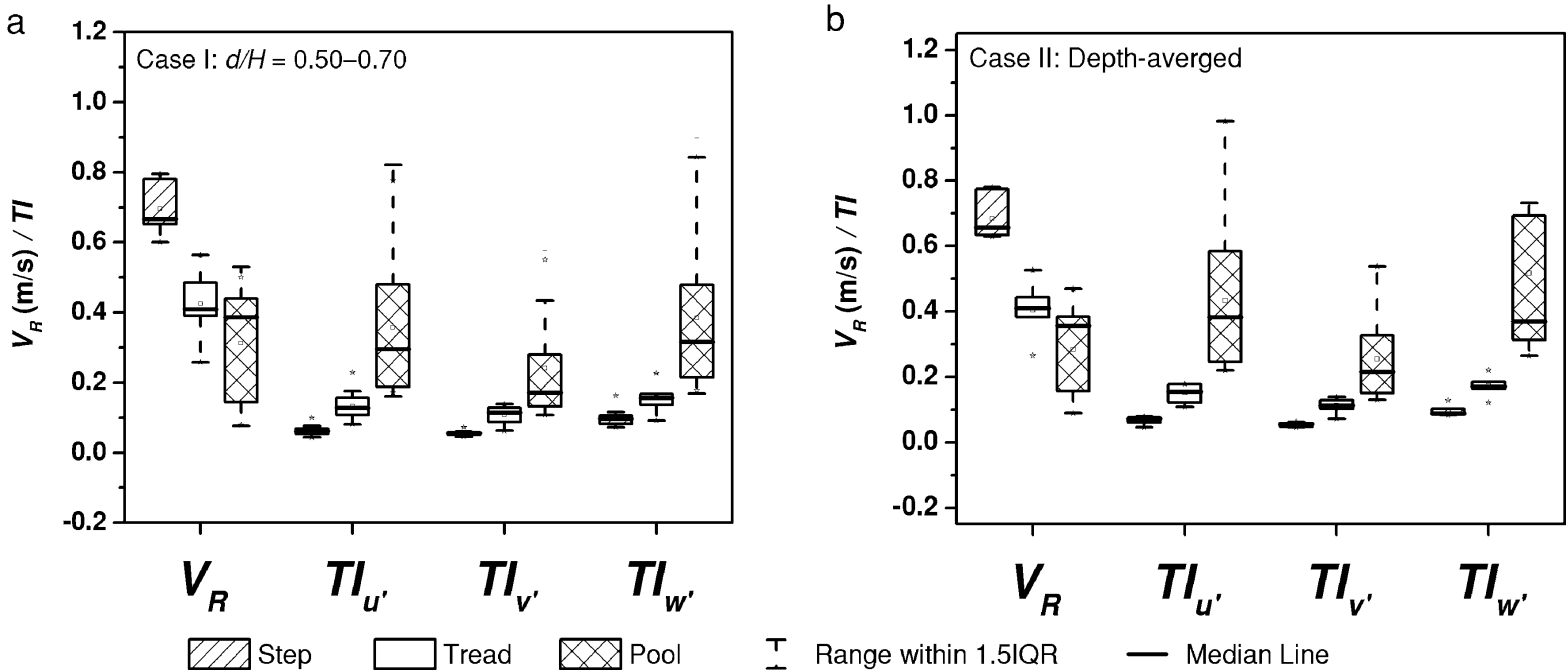
Distribution of resultant velocity magnitude *V*_*R*_ and turbulence intensities *TI* of the fluctuating velocities, *u*′, *v*′, and *w*′ for different morphological unit: (**a**) Case I: data points confined to *d*/*H* = 0.50–0.70. (**b**) Case II: depth-averaged values at each vertical.

To examine the statistical differences in the distribution of velocity and turbulence intensities across the morphological units, the 24 vertical velocity measurements comprising longitudinal and cross-stream points have been subjected to Kruskal–Wallis ANOVA. The earlier studies that sought to distinguish the morphological units on the basis of velocity components performed one-way ANOVA on the datasets. Although the measurements on steps, treads and pools are independent of each other and randomly sampled, the available data fails to uniformly conform to normal distribution, which is a prerequisite for ANOVA test. Therefore, the present work revisits this analysis for velocity components, velocity magnitude and turbulence intensities using Kruskal–Wallis ANOVA which is a non-parametric test that does not assume a normally distributed dataset. The analysis is conducted on the ranks of the data values rather than the data values, and tests whether the median values are significantly different from each other. A resultant *p* value of 0 from the analysis indicates that there is significant difference between the groups, while a *p* value of 1 indicates vice-versa. The null hypothesis of Kruskal–Wallis ANOVA is that the sample groups come from the same population. Closeness of the *p* value to 0 is a measure of the confidence in rejecting the null hypothesis. In the present study, data points were categorized with respect to *d*/*H* values to normalize the effect of depth on the velocity variations, and the data points confined in the range *d*/*H* = 0.50–0.70 were considered for the test (Case I). The *d*/*H* is thus selected to obtain a wider range of data points pertaining to the average velocity which is typically at *d*/*H* = 0.6. The non-parametric test was also repeated for depth averaged values (Case II) to produce similar results. Except in the case of cross-stream velocity *v*, all other groupings showed significant difference between the median values for step, tread and pool data points. The *p* values of 0.24 and 0.41 were obtained for the hypothesis test on *v* for Case I and Case II, respectively. This shows that the variation in the cross-stream velocity is independent of the morphological type and is not a characteristic feature of step-pool system in a straight channel. The step-pool system that encounters bends within the reach may have an influence on the cross-stream velocity component. The results of the statistical analysis and box-plots of the velocity magnitude and turbulence intensities for both Cases I and II are given in Table 2 and Fig. 3, respectively. A negligible absolute error of 0.027 m/s and 0.031 m/s in velocity magnitude was obtained between the mean and median of Cases I and II, respectively. Whereas, a maximum absolute error of 0.134 and 0.087 was observed in the respective turbulence intensities. However, the differences in the methods are not substantial enough to alter the results of the hypothesis testing.

**Table 2:** Comparison and analysis results of Kruskal–Wallis ANOVA for data points in the range of *d*/*H* = 0.50–0.70 and depth-averaged values at various verticals across the morphological units.

	*V*_*R*_			*TI*_*u*′_			*TI*_*v*′_			*TI*_*w*′_		
	Step	Tread	Pool	Step	Tread	Pool	Step	Tread	Pool	Step	Tread	Pool
**Case I: *d*/*H* = 0.50–0.70**												
Mean	0.70	0.43	0.31	0.06	0.13	0.36	0.06	0.11	0.24	0.1	0.16	0.38
Median	0.68	0.41	0.39	0.06	0.13	0.30	0.06	0.11	0.17	0.10	0.16	0.32
*p* value	9.43E−14			4.55E−18			7.95E−18			5.94E−18		
**Case II: Depth-averaged**												
Mean	0.69	0.40	0.29	0.07	0.15	0.43	0.06	0.11	0.26	0.10	0.17	0.52
Median	0.66	0.41	0.36	0.07	0.16	0.38	0.06	0.11	0.22	0.09	0.17	0.37
*p* value	1.91E−04			2.84E−05			3.93E−05			3.22E−05

**Figure 4:**
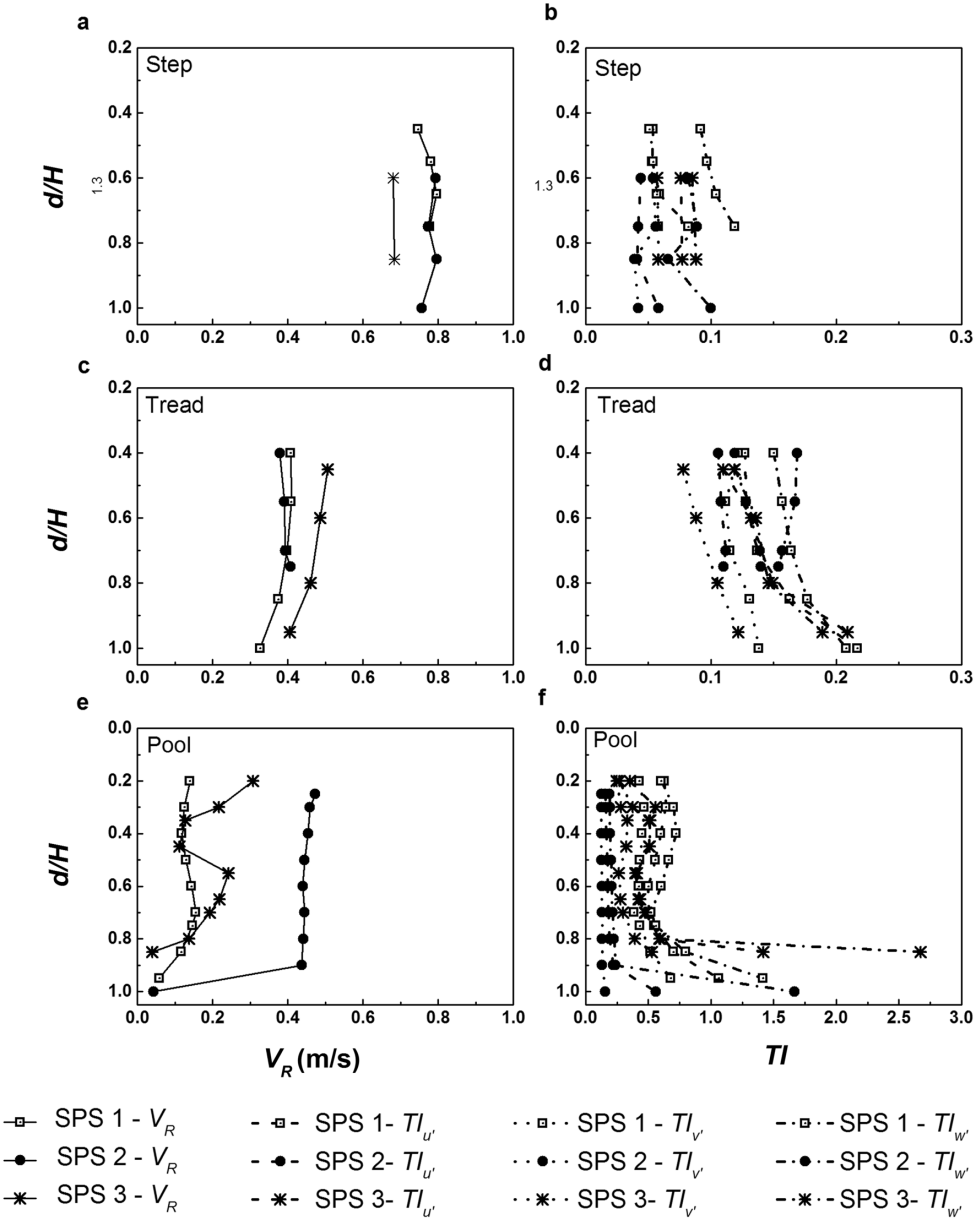
Variation of resultant velocity magnitude *V*_*R*_ and turbulence intensities *TI* of the fluctuating velocities, *u*′, *v*′, and *w*′ along the depth: (**a**) *V*_*R*_ at Step, (**b**) *TI* at Step, (**c**) *V*_*R*_ at Tread, (**d**) *TI* at Tread, (**e**) *V*_*R*_ at Pool, and (**f**) *TI* at Pool.

The average values of *TI*_*u*′_, *TI*_*v*′_, and *TI*_*w*′_ combining the 24 verticals (*d*/*H* ranging from 0.20 to 1.00) are 0.065,0.055 and 0.097 for steps, 0.146, 0.110, and 0.165 for treads, and 0.453, 0.265, and 0.523 for pools, respectively. The values show an increase of 55%, 50% and 41% for *TI*_*v*′_ with respect to *TI*_*u*′_ for step, tread and pool, respectively, while a sizeable increase of 597%, 382% and 439% for *TI*_*w*′_ with respect to *TI*_*u*′_, which evidently indicates the dominance of vertical fluctuations in the pools. The pattern of variation of turbulence intensities at step, tread and pools can be better understood with the help of vertical profiles. Figure 4 shows the vertical profiles of velocity magnitude and turbulence intensity profiles corresponding to step, tread and pool regions in different step-pool systems, namely, SPS 1, SPS 2, and SPS 3 (see “Methods”). Compared to the velocity profiles in step and tread, a visible mid-profile shear layer can be seen in the pool. Previous researchers have identified the presence of mid-profile shear in regions of wake turbulence. Thompson and Wohl^4^ illustrated the shear layer downstream of steps with the help of velocity profiles in step-pool systems. Baki et al.^37^ illustrated the presence of shear layer in the wake turbulence regions of a rock-ramp fish pass. A staggered arrangement of natural boulders of equivalent diameter 14 cm was used to prepare the rock-ramp bed. The wake area downstream of the boulders is similar to the downstream of steps in step-pool systems. Fang et al.^38^ illustrated the shift in the vertical profile of Reynolds shear stress due to near-bed and boulder-induced shear stresses. In the present study, the shear layer is prominent in SPS 3, milder in SPS 1 and fairly non-existent in SPS 2 which corresponds to the profile at *X* = 2.40 m in Fig. 2. The shear layer is generated due to the momentum exchange occurring in the pools consequent to flow impingement. The occurrence of the shear layer and the magnitude of velocity shift depend on the characteristics of the upstream step unit and spacing between the upstream step and point of interest. In the case of leaky step units where some portion of the step cross section is devoid of elevated step units, the flow passes through without causing consideration impingement to the downstream pool. Hence, the flow does not produce a downstream wake region resulting in the absence of shear layers in the vertical profile. The same can be observed from Fig. 4e, where the vertical section in SPS 2 existed downstream of a leaky step unit, which also lead to lower levels of energy dissipation and less velocity reduction.

The magnitude of turbulence intensities is lower on step and tread, and maximum in the pools as can be observed in Fig. 4b, d and f, respectively. The fluctuations are higher in the pools due to the varied velocity distribution and wake turbulence characteristics in the pools.

### Reynolds shear stress

**Figure 5:**
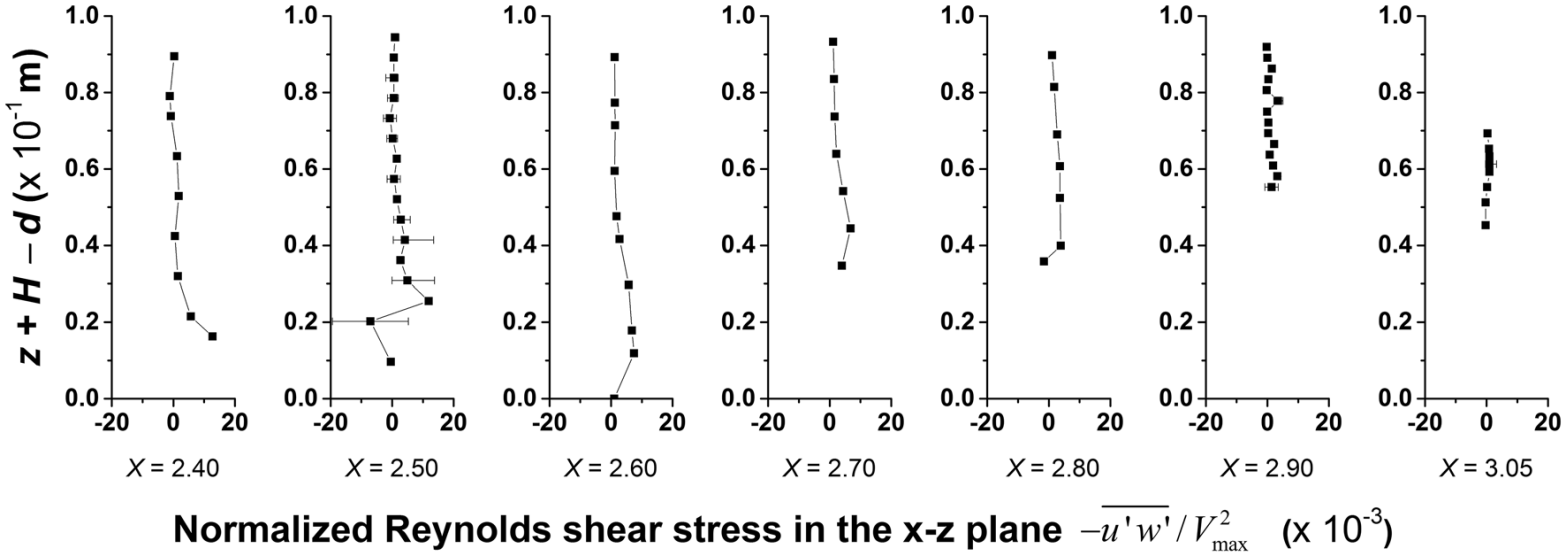
Variation of time averaged and normalized Reynolds shear stress in the *x–z* plane $( - \overline{{u^{{\prime }} w^{{\prime }} }} /V_{\max }^{2} )$ in the longitudinal direction of SPS 2 (*V*_*max*_ = 0.796 m/s).

Reynolds shear stress is the stress generated due to the momentum exchange between the fluctuating velocity components. The range of shear stress in the flow medium has implications in the suitability of a flow body to various aquatic lives since high levels of shear stress may even lead to major injuries or mortality to the species. The vertical profiles of the time averaged and normalized Reynolds shear stress in the *x*–*z* plane in the longitudinal direction is shown in Fig. 5. The *x*-axis shows the normalized Reynolds shear stress $- \overline{{u^{{\prime }} w^{{\prime }} }} /V_{\max }^{2}$ for each section, where *V*_*max*_ = 0.796 m/s is the maximum velocity measured during the experimental runs. The variable on the *y*-axis follows the same convention as described in Fig. 2. The fluctuations in the profile are more in the deeper locations in the pool (*X* = 2.40 m to 2.80 m) due to the increased turbulence at the bottom as a result of flow impingement. The error bar shown for X = 2.50, 2.90 and 3.05 is calculated from the additional 4 verticals measured in the cross-stream direction, for each of the sections. The maximum Reynolds shear stress variation is observed in *x*–*z* plane compared to *x*–*y* and *y*–*z* planes, with normalized values ranging from − 19.477 to 13.729. The absolute maximum value of 19.477 amounts to 12.34 N/m^2^ in model scale and 40.73 N/m^2^ in prototype scale. Reynolds shear stress as low as 30 N/m^2^ can cause reduction in startle response in some species^39^. Therefore, ensuring acceptable limits of turbulence fluctuations is essential in the design for artificial constructions of the step-pool morphology. While recreating the morphology for a fish pass design, control can be placed on the pool volume, characteristic grain size (equivalent to step height) or allowable discharge to reduce the turbulence levels in pools. However, this entails detailed study into the cause and effect of these parameters on the hydrodynamics.

### Turbulent kinetic energy

**Figure 6:**
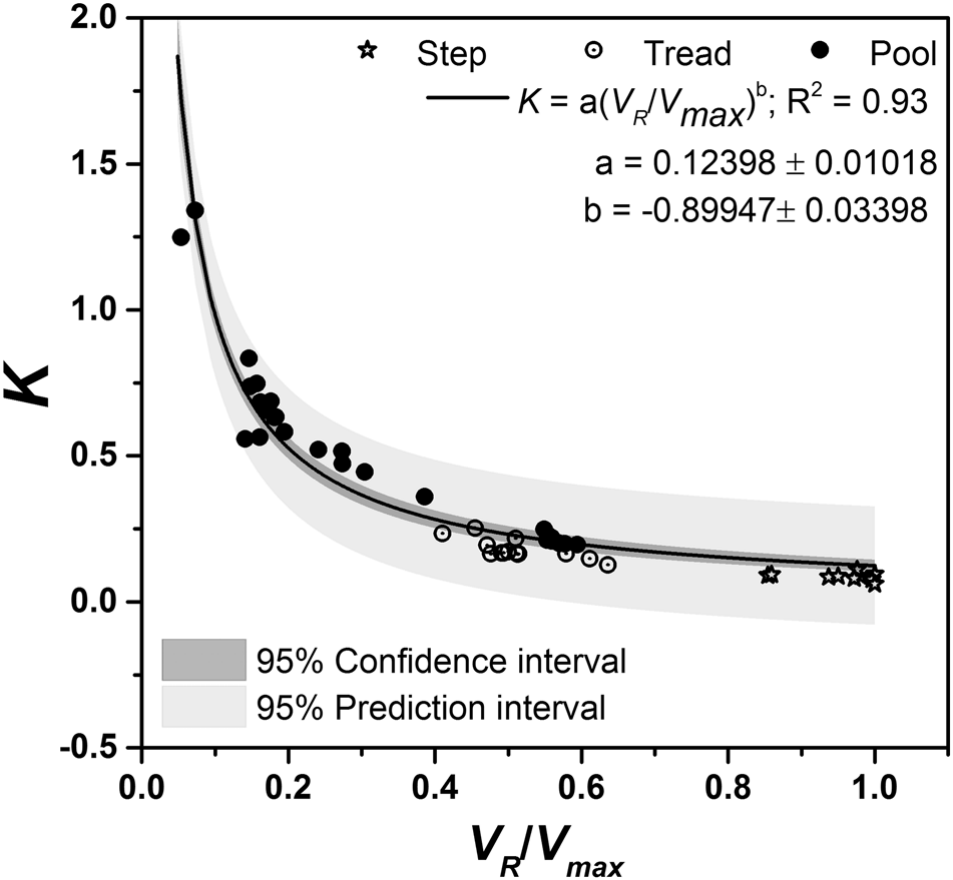
Variation of normalized turbulent kinetic energy *K* with the time-averaged thalweg velocity ratio *V*_*R*_*/V*_*max*_ (*V*_*max*_ = 0.796 m/s).

Another indicator of turbulence characteristic to the morphological units in the present study is Turbulent Kinetic Energy (TKE) which is a measure of kinetic energy per unit mass of the turbulent flow. It is an important parameter that determines the locomotive characteristics of various species^40^ and key to evaluating the energy loss to fishes^41^. In the present study, normalized form of turbulent kinetic energy (*K*) follows an inverse power relation to the velocity magnitude as shown in Fig. 6. The *x*-axis is normalized using *V*_*max*_ and TKE is normalized by the transformation $K = \sqrt {{\text{TKE}}} /V_{R}$. The data is inclusive of all the depth-wise data points measured over step, tread and pool regions along the thalweg. Larger values of *K* occurred in pools followed by tread and step regions. The pattern is comprehensible from visual observation of the flow field, where the flow occurs as high-velocity sheet with limited agitation over tread and step, resulting in plunging flow with recirculating eddies in the pools. A non-linear curve fitting of type Power-Allometric 1 was used to generate the empirical equation $K = a\left( {{{V_{R} } \mathord{\left/ {\vphantom {{V_{R} } {V_{\max } }}} \right. \kern-\nulldelimiterspace} {V_{\max } }}} \right)^{b}$, where *a* = 0.12398 and *b* = − 0.89947 with a standard error of ± 0.01018 and ± 0.03398, respectively. The coefficient of determination of the plot is 0.93.

### Energy dissipation factor

The energy dissipation factor (*EDF*) is an engineering design parameter that checks the turbulence level in the fish pathways. The flow energy must be sufficiently dissipated to ensure velocity levels less than 2 m/s. The *EDF* is also representative of the eddies and turbulence generated due to flow impingement into pools. Contrary to the conventional pool fish passes and slot fish passes, the pool cross-sections in natural step-pools are not uniform^33^. The pool dimensions vary along the reach and accordingly reflects in the *EDF* values. Hence, calculation of *EDF* using the equation $EDF=\gamma QS/A$ would result in considerable errors since A is not a constant within and across step-pool systems, where γ is the specific weight of water, *Q* is the discharge, *S* is the bed slope and *A* is the cross-sectional area of the pool. Here, the *EDF* calculations were based on the basic equation $EDF = \gamma Q\Delta H/\forall$, where ΔH is the drop in water elevation level per pool and $\forall$ is the pool volume. The step-pool systems 1, 2 and 3 have been evaluated for *EDF*. The pool volume was calculated applying the trapezoidal rule to the wetted areas of cross-sections in pool spaced at 10 cm apart. The wetted area was calculated from the measured bed and water elevation levels. The region starting immediately downstream of the steps up to the exit slope of the scour pool is considered for calculating the pool volume. Table 3 presents the pool dimensions and *EDF* values obtained in the present study. The *EDF* values obtained for step-pool systems 1, 2, and 3 were 321, 207, and 123 W/m^3^ in model scale, respectively. The results corresponds to 590, 380, and 226 W/m^3^ in prototype scale. However, a value of 150 W/m^3^ should not be exceeded to ensure acceptable levels of turbulence in the pools^33^. Specific *EDF* criteria for various fish species are available to design fish passes accommodating the requirements of the predominant fish population^42^.

**Table 3:** Computation of energy dissipation factor in step-pool systems 1, 2 and 3.

Location	*Q* (m^3^/s)	*ΔH* (m)	$\forall$ (m^3^)	*EDF* (W/m^3^) Model scale	*EDF* (W/m^3^) Prototype scale
SPS 1	0.021	0.0609	0.037178	321.39	590.25
SPS 2	0.021	0.0381	0.036137	206.86	379.90
SPS 3	0.021	0.0239	0.038142	122.94	225.79

Considering the range of shear stresses and energy dissipation factors obtained in the present study, it can be inferred that construction of step-pool fish passes simulating the field parameters may not provide adequate flow conditions for a step-pool type fish pass. For the design of step-pool type fish passes, the pool volumes should be back calculated from the *EDF* equation for specific species. The translation of the pool volume in terms of the width of channel, pool length and pool depth will ensure lower levels of turbulence intensities and shear stresses in the passage. Since the interest towards close-to nature fish passes have been developed only in the recent years, specific guidelines for step-pool type fish passes are yet to be formulated. This calls for research in artificial step-pool constructions based on the pool and turbulence requirements of the dominant target species. Nevertheless, the nature of hydrodynamics in the self-formed and artificially constructed would coincide since the step-pool bed morphology has an inherent tendency to attain a state of maximum resistance. The concept of creating artificial structures is limited to providing and placing the bed materials into required bed slopes and approximate design dimensions. The ultimate bed morphology of the structure will be modelled over time by the hydraulic force of the flowing water.

## Conclusions

Step-pools are characterized by unique morphological units and spatially varying hydrodynamics. In recent times, applications of step-pool morphology in the design of close-to-nature step-pool fish passes and conveyance systems are gaining popularity considering ecological and economic benefits. Better understanding about the flow characteristics in a self-formed step-pool will aid in the design of artificial fish structures and in improving hydraulic models of varied applications. The present study deals with the analysis of flow turbulence over the different morphological units such as step, tread and pool. Laboratory experiments were conducted on a physical model of scale ratio 3.3:1 downscaled from field investigations carried out in the southern ranges of Western Ghats, India. Velocity data collection involved the measurement of 24 vertical velocity profiles in the longitudinal and transverse directions across three sets of step-pool systems. The present study proposes an empirical equation of the form Power-Allometric1 to relate normalized turbulent kinetic energy to the velocity magnitude. Evaluation of velocity distribution in step, tread and pool regions using Kruskal–Wallis ANOVA showed significant differences between the morphological units. Hence, meso-scale analysis of hydrodynamics is required to efficiently reproduce the step-pool morphology for artificial constructions. The average values of *TI*_*u*′_, *TI*_*v*′_, and *TI*_*w*′_ combining the 24 verticals is 0.065, 0.055 and 0.097 for steps, 0.146, 0.110, and 0.165 for treads, and 0.453, 0.265, and 0.523 for pools, respectively. Where, *TI*_*u*′_, *TI*_*v*′_, and *TI*_*w*′_ are the turbulence intensities of the fluctuating velocity components *u*′, *v*′, and *w*′, respectively. The normalized values of Reynolds shear stress in the *x*–z plane ranged from − 19.477 to 13.729 indicating higher levels of turbulence in the deeper pool region, which would result in unsuitable living environment to the benthic invertebrates. In addition, energy dissipation factors obtained for the three step-pool systems, 321, 207, and 123 W/m^3^ reveal insufficient pool volume for energy dissipation. It is inferred from the present study that creation of a step-pool fish pass simulating parameters existent in the field do not warrant an efficient fish pass. The pool dimensions have to be designed based on the energy dissipation factors required as per species criteria. Guidelines for the design of close-to-nature step-pool fish passes can be developed only after conducting iterative model study.

## Methods

This section describes the methods of data collection in the field site and laboratory setup. The approaches adopted for the calculation of various parameters, presentation and results are also explained.

### Field study

We collected field data from the southern part of Western Ghats in Peninsular India. The mountain ranges in the Western Ghats are among the eight hottest hotspots of biodiversity in the world, as ranked by UNESCO. Our study was restricted to a 100 m stretch of step-pool stream, carried out during July and August months of 2019 in Erumakolli, Meppadi Panchayat, Wayanad, Kerala, India. It involved the measurements of streambed topography, streambed material size, and point velocity in the stream.

We obtained the bed material sizes based on the Wolman method, which comprises the measurement of intermediate axis of 100-bed materials by pacing along the study reach^43^. This method is suitable for listing bed materials greater than 2 mm in size and can be done even with live flow conditions. The characteristic bed material size in step-pool mountain streams is represented by *D*_84_ since it is in the same order of the step heights. Where, *D*_84_ represents the size coarser than 84% of the measured sizes. The value of *D*_84_ was obtained as 430 mm from the frequency distribution curve of the bed material sizes.

The streambed topography was measured using a Total station Leica TS 06R with 5-s accuracy. The thalweg points along the reach were measured at 1 m interval to obtain the average bed slope. Measurements were taken in the cross-stream directions as well for sections representative of step, tread and pool morphologies. The average bed slope of the reach is 6.7%, and the average stream width is 3.3 m.

Point velocity measurements were captured using Sontek FlowTracker2, an Acoustic Doppler Velocimeter (ADV) suited for field-flow conditions. It consists of a side looking probe with a transmitter and two receivers collecting two-dimensional velocity data at 2 Hz frequency. The dynamic range of the instrument as specified by the Manufacturer is ± 1% of measured velocity + 0.25 cm/s. Earlier field study in step-pool stream reported in the literature used a FlowTracker with 1 Hz sampling frequency for a duration of 180 seconds^35,36^. The same measurement duration was adopted in the present study, after verification with stationarity analysis. The sample volume is cylindrical with a diameter of 6 mm and a height of 9 mm, spaced 10 cm away from the transmitter axis. A recorded sample value is the average of 20 pings of measurements. The time-series data obtained from the instrument is filtered for Sound to Noise Ratio (SNR) > 10 and for outliers greater than two times the interquartile range^44^. The resulting data sets are evaluated for its root mean square error of the fluctuating velocity components, *u′*_*rms*_ and *v′*_*rms*_. Its non-dimensional form is obtained by dividing *u′*_*rms*_ and *v′*_*rms*_ with velocity magnitude, generally termed as Turbulence Intensities *TI*_*u′*_ and *TI*_*v′*_, respectively. Prior to further analysis, the dataset is filtered for *TI* values greater than two since it denotes large scatter of the velocity values which is unreliable due to the low sampling frequency. All of the removed data with *TI* values greater than two, belonged to the base of step location where flow plunging and recirculation is more. Therefore, velocity values at the base of steps have not been included for further analysis in this study. The resulting data comprising steps, treads and pool sections summed up to 20 cross-sections (see Supplementary Table S1). Owing to the low sampling frequency of the instrument, only the velocity values have been used for validation of the experimental setup.

We measured the discharge using the same instrument at a prismatic section of a bridge culvert located upstream of the study reach. The values of flow discharges are directly obtained from the instrument after calculation using the mid-section method. The flows that occurred during the study period were 0.1, 0.2, 0.3 and 0.4 m^3^/s.

### Experimental study

The experimental setup was constructed in the Hydraulics Laboratory, Indian Institute of Technology Madras, India. It consists of an inlet tank, an ensuing rectangular flume of dimensions 11 m × 1 m × 1 m and an exit tank. The channel is glass-walled from a distance of 3.5 m and up to a distance of 9.7 m from the inlet tank. The flow through the flume is re-circulated with the help of a 30 Hp centrifugal pump and sump arrangement. The flow is regulated using a Supervisory Control and Data Acquisition (SCADA) system and verified with an inline electromagnetic flow meter MagFlow 6410, accurate to ± 0.5% of the measured value.

A scale ratio of 3.3:1 was chosen to prepare an undistorted model of the step-pool sequence maintaining dynamic similarity between the field and model using Froude’s Model Law. The independent variables that have been scaled down are stream width, bed material size and discharge. The bed material sizes in the present study vary between 6 and 155 mm, which are scaled down from the bed material sizes measured from the field. A plain bed of slope 6.7% was prepared initially with a uniform mix of bed materials. Step-forming bed materials (size ranging from 76.2 to 155 mm) have been randomly clustered with a clear spacing of 1 m in the flume, to aid the formation of the step-pool sequence. The chosen spacing is approximately equal to the average step-to-step distance of 3.5 m measured in the field. A high flow of 70 Lps (1.39 m^3^/s in prototype scale) was arbitrarily chosen as the formative discharge, which is the discharge that forms and stabilizes the bed morphology. Any discharge lower than the formative discharge would not destabilize the bed surface. The procedure for the bed preparation followed the works of Comiti et al.^45^ that examined the bed morphology and flow resistance in self-formed step-pool channels.

A photograph of the step-pool sequence formed consequent to the formative discharge is shown in Fig. 1d. The bed measurements are taken at 5 cm spacing along the thalweg using a digital point gauge of resolution 0.01 cm. The surficial bed distribution is obtained with the help of ImageJ, a freely available image processing and analysis tool developed by the National Institutes of Health (NIH), US. Photographs of the bed surface are captured and loaded into ImageJ and the scale of the photograph was set against an object of known measurement placed in the same plane. A manual method of measurement is selected wherein each bed particle along the identified thalweg is individually specified by the user. The automated measurement method was not adopted due to the difficulty in identifying the boundaries of separate bed materials owing to the closely packed bed structure and non-uniform color on each bed material. In order to apply the automated measurement method of ImageJ, it would require coloring of different classes of bed material sizes for easy identification. The characteristic bed material size *D*_84_ and the average bed slope *S* is obtained as 132 mm (436 mm in prototype scale) and 6.42%, respectively.

The flow in the experimental setup corresponding to the flows observed in the field range from 0.005 to 0.02 m^3^/s. To ascertain sufficient flow depth in the flume for velocity measurement, a flow of 0.02 m^3^/s have been chosen for the physical model study. The flow velocity was measured using Nortek Vectrino, a 3D-ADV with a side-looking probe. It consists of a 10 MHz transmitter and four receivers that collect samples at 25 Hz frequency. The sampling volume of diameter 6 mm and height 5.5 mm (user-defined) is located at 5 cm distance from the transmitter. The dynamic range of the instrument as specified by the Manufacturer is ± 1% of the measured value ± 1 mm/s. A velocity range of ± 1 m/s has been selected throughout the experiment to prevent velocity aliasing. In highly turbulent flow fields such as in step-pools, a correlation coefficient as less as 40% is acceptable if at least 70% of the raw data is retained after filtration^46^. Therefore, the raw ADV data was filtered for SNR > 15 dB and correlation > 40% (subjected to a minimum of 70% good data) using Win-ADV, which is a widely used software for viewing and post-processing of ADV files^47–49^. The subsequent data was processed by applying the phase-space threshold de-spiking method of Goring and Nikora^50^ which is inbuilt in the software. However, the removed data have not been replaced since replacement would result in additional spikes in turbulent flows^51^. The scope of the present study is limited to the mean flow statistics such as velocity magnitude, turbulent kinetic energy and Reynolds stresses. Data replacement would be required if one is interested in the spectral analysis of the velocity fluctuations.

**Figure 7:**
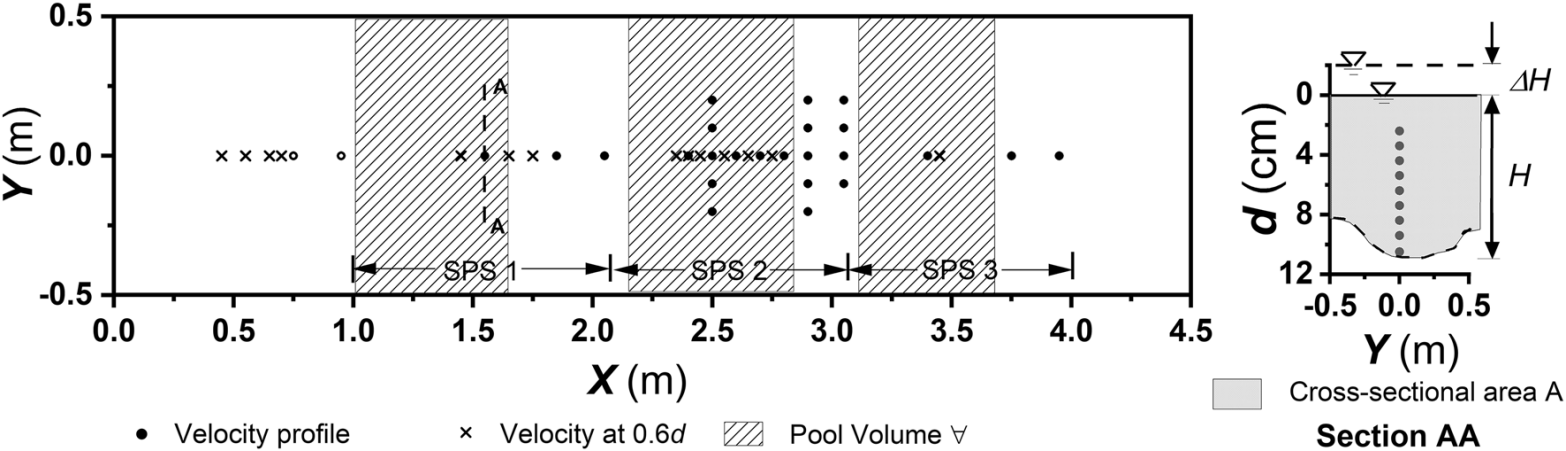
Illustration of velocity measurement points in the experimental setup.

The laboratory flow measurement consists of (i) point velocity measurements along the flow thalweg at *d* = 0.6*H* with roughly 10 cm spacing between the sections, where *d* is the depth of measurement and *H* is the total flow depth at a particular point in the flume bed, (ii) vertical velocity profiles at representative thalweg points on step, tread and pool in consecutive step-pool systems (SPS) 1, 2 and 3 as shown in Fig. 7, (iii) the propagation of vertical velocity profile in SPS 2, and (iv) vertical velocity profiles in the cross-stream direction for representative step, tread and pool sections in SPS 2.

For the vertical velocity profile, the top most data point was at 2.4 cm below the water surface since the receivers had to be completely immersed in the water to get 3-D data. The remaining data points were spaced at 1 cm along the depth up to the lowest point attainable considering the uneven bed topography. Although data collection included sections pertaining to base of steps, entire data failed to pass the filtration and data processing. The final set of good data amounts to 30 thalweg velocity points at *d* = 0.6*H* and 24 vertical profiles (see Supplementary Table S2). Less than 5% of the velocity time-series data have been removed for 64% of the total data points, and more than 85% of the data have been retained for 96% of the points.

### Flow and turbulence parameters

Three-dimensional flow velocity is obtained from Nortek Vectrino ADV. The stream-wise velocity component *u*, the cross-stream velocity component *v*, and the vertical velocity component *w* is positive in the flow direction, towards the left-side of flow, and towards the water surface, respectively. The respective fluctuating components are represented by *u*′, *v*′, and *w*′. The resultant velocity magnitude is represented as *V*_*R*_. The turbulence intensities *TI*_*u*′_, *TI*_*v*′_, *TI*_*w*′_ are obtained by dividing the root mean square of the fluctuating velocity components, *u′*_*rms*_, *v′*_*rms*_ and *w′*_*rms*_, by the flow magnitude *V*_*R*_ ($TI_{{u^{{\prime }} }} = \, u_{rms}^{{\prime }} /V_{R}$_,_ $TI_{{v^{{\prime }} }} = v_{rms}^{{\prime }} /V_{R}$ and $TI_{{w^{{\prime }} }} = w_{rms}^{{\prime }} /V_{R}$). Turbulent kinetic energy (TKE) can be calculated as ${\text{TKE}} = 0.5\rho \left[ {\left( {u_{rms}^{{\prime }} } \right)^{2} + \left( {v_{rms}^{{\prime }} } \right)^{2} + \left( {w_{rms}^{{\prime }} } \right)^{2} } \right]$, where *ρ* is the density of water. The normalized form of turbulent kinetic energy (*K*) obtained as $K = \sqrt {TKE} /V_{R}$ is used for presenting the results. The energy dissipation factor defined for the fish passage is $EDF = \gamma Q\Delta H/\forall$ which could be redefined as $EDF = \gamma QS/A$, where *Q* is the flow rate, *ΔH* is he elevation difference between consecutive pools, $\forall$ is the pool volume, γ is the specific weight of water, *S* is the bed slope, and *A* is the pool sectional area^52^.

## Supplementary Information

Supplementary Table S1.

Supplementary Table S2.
